# Cardiovascular magnetic resonance in the identification of extra-cardiac causes of myocarditis: a case series

**DOI:** 10.1093/ehjcr/ytae232

**Published:** 2024-05-06

**Authors:** Hichem Sakhi, Guillaume Reverdito, Gilles Soulat, Elie Mousseaux

**Affiliations:** AP-HP, Hôpital Européen Georges-Pompidou, 20 rue Leblanc, 75015 Paris, France; AP-HP, Hôpital Européen Georges-Pompidou, 20 rue Leblanc, 75015 Paris, France; Institut National de la Santé et de la Recherche Médicale, PARCC, Paris, France; Université de Paris-Cité, 75015 Paris, France; AP-HP, Hôpital Européen Georges-Pompidou, 20 rue Leblanc, 75015 Paris, France; Institut National de la Santé et de la Recherche Médicale, PARCC, Paris, France; Université de Paris-Cité, 75015 Paris, France; AP-HP, Hôpital Européen Georges-Pompidou, 20 rue Leblanc, 75015 Paris, France; Institut National de la Santé et de la Recherche Médicale, PARCC, Paris, France; Université de Paris-Cité, 75015 Paris, France

**Keywords:** Myocarditis, Cardiovascular imaging, Cardiovascular magnetic resonance imaging, Case series, Infectious diseases, Cancer

## Abstract

**Background:**

Myocarditis is challenging to diagnose due to its varied presentations. Endomyocardial biopsy is the gold standard for diagnosis, but its invasive nature has led to alternative non-invasive modalities, notably cardiovascular magnetic resonance (CMR). Identifying the precise aetiology of myocarditis is crucial for effective treatment, yet extra-cardiac causes are often overlooked. In this paper, we spotlight the underexplored role of CMR in diagnosing extra-cardiac aetiologies, utilizing three insightful cases for illustration.

**Case summary:**

The first case is a 31-year-old patient with myocarditis secondary to a pyogenic liver abscess, identified through CMR, who improved after abscess drainage. The second case involves a 54-year-old patient with myocarditis attributed to adult T-cell leukaemia–lymphoma, with the loco-regional thickening process identified thanks to CMR. This patient had an unfavourable disease progression due to the underlying malignancy. The third case concerns a 23-year-old patient suffering from myocarditis associated with pneumonia, again illustrated effectively through CMR imaging, who recovered after antibiotic treatment.

**Discussion:**

These cases underline the overlooked potential of CMR in diagnosing extra-cardiac aetiologies of myocarditis, even though such causes are rare. Despite current guidelines recognizing the importance of identifying the aetiology of myocarditis, they do not explicitly address the role of CMR in diagnosing extra-cardiac aetiologies. This article, therefore, proposes that future guidelines could emphasize the utility of CMR in exploring these causes, potentially leading to more accurate diagnoses and improved patient outcomes. It also advocates for a comprehensive, multidisciplinary approach to myocarditis diagnosis, encouraging vigilance for potential loco-regional causes, and calls for further research in this area.

**Learning points**

- These cases highlight the pivotal role of cardiovascular magnetic resonance in diagnosing loco-regional aetiologies of myocarditis, showcasing its capacity to uncover non-traditional causes.
- The diversity of aetiologies of myocarditis, as illustrated in these cases, underscores the importance of a comprehensive, multidisciplinary approach in diagnosis and treatment. Despite the rarity of extra-cardiac causes, clinicians should remain vigilant and consider these potential causes in their diagnostic processes to ensure optimal patient outcomes.

## Background

Myocarditis, a common source of acute myocardial injury, arises from a range of inflammatory factors, with the most prevalent being viral infections.^1^ Its pathophysiological mechanisms encompass both direct viral damage and subsequent immune-mediated responses.^2^ Diagnosing myocarditis, however, has been a significant challenge in clinical practice due to its diverse presentations, from asymptomatic patients to severe cardiac dysfunction and arrhythmias. Traditionally, endomyocardial biopsy is the gold standard for diagnosing myocarditis.^3,4^ However, its invasive nature, potential complications, and sampling errors have called for alternative non-invasive diagnostic modalities.^5^ Cardiovascular magnetic resonance (CMR), given its capability for myocardial tissue characterization, has taken centre stage as a pivotal tool in diagnosing myocarditis. As per the 2018 updated Lake Louise Criteria, CMR stands as a preferred non-invasive alternative for detecting acute myocardial inflammation.^6,7^ The diagnosis of myocarditis according to these criteria relies on the presence of both a T2 criterion (global or regional increase of myocardial T2 relaxation time or an increased signal intensity in T2-weighted CMR images) and a T1 criterion [increased myocardial T1, extracellular volume, or late gadolinium enhancement (LGE)]. However, despite the advances in imaging techniques, determining the specific aetiology of myocarditis remains challenging. This diagnostic gap is particularly significant since different aetiologies may require varying treatment strategies and affect patient prognosis.^4^ The loco-regional causes of myocarditis are scantily described in both the literature and current guidelines. Here, through three cases, we illustrate the unexplored potential of CMR in diagnosing extra-cardiac aetiologies of myocarditis, which, despite their rarity, may have a substantial therapeutic impact.

## Summary figure

**Table:** 

Timeline	
Patient 1	
Day 1	Development of fever, abdominal and chest pain.
Day 2	Admission to emergency department.
	Troponin elevation (30-fold the upper normal value).
	Transfer to cardiac intensive care unit.
Day 4	Cardiovascular magnetic resonance (CMR): T2 mapping and subepicardial late gadolinium enhancement (LGE) in the inferolateral region. Visualization of liver abscess.
Day 5	Computed tomography: confirmation of liver abscess.
	Percutaneous liver abscess drainage.
Day 10	Symptoms relief and discharge.
Day 180	Follow-up CMR: complete abscess regression, normalization of T2 mapping but persistent LGE scar.
Patient 2	
Day 1	Diagnosis of adult T-cell leukaemia/lymphoma.
Day1–Day 210	Six rounds of chemotherapy CHOEP (cyclophosphamide, doxorubicin, etoposide, vincristine, and prednisone).
	Two rounds of high-dose methotrexate chemotherapy treatment.
	Tumoural progression with peritoneal carcinomatosis.
Day 225	Chest pain with significant troponin elevation (28-fold the upper normal value).
	CMR: T2 mapping and subepicardial LGE in the inferolateral region. Loco-regional peri-splenic, peritoneal, and diaphragmatic hyperintense thickening process due to peritoneal lymphoma carcinomatosis in contact of the heart.
Day 240	Deterioration of her general condition and poor prognosis of the disease with lack of therapeutic options.
	Initiation of palliative care.
Patient 3	
Day 1	Cough, fever.
Day 5	Oppressive retrosternal chest pain.
	Admission to the emergency department.
	Troponin elevation (315-fold the upper normal value).
	Transfer to cardiac intensive care unit.
Day 6	CMR: T2 mapping and subepicardial LGE in the inferolateral region. Visualization of pneumonia.
	Computed tomography: pneumoniae confirmed.
	Antibiotic treatment.
Day 11	Symptoms relief and discharge.
Day 180	Follow-up CMR: normalization of T2 mapping but persistent LGE scar.

### Patient 1

**Figure 1:**
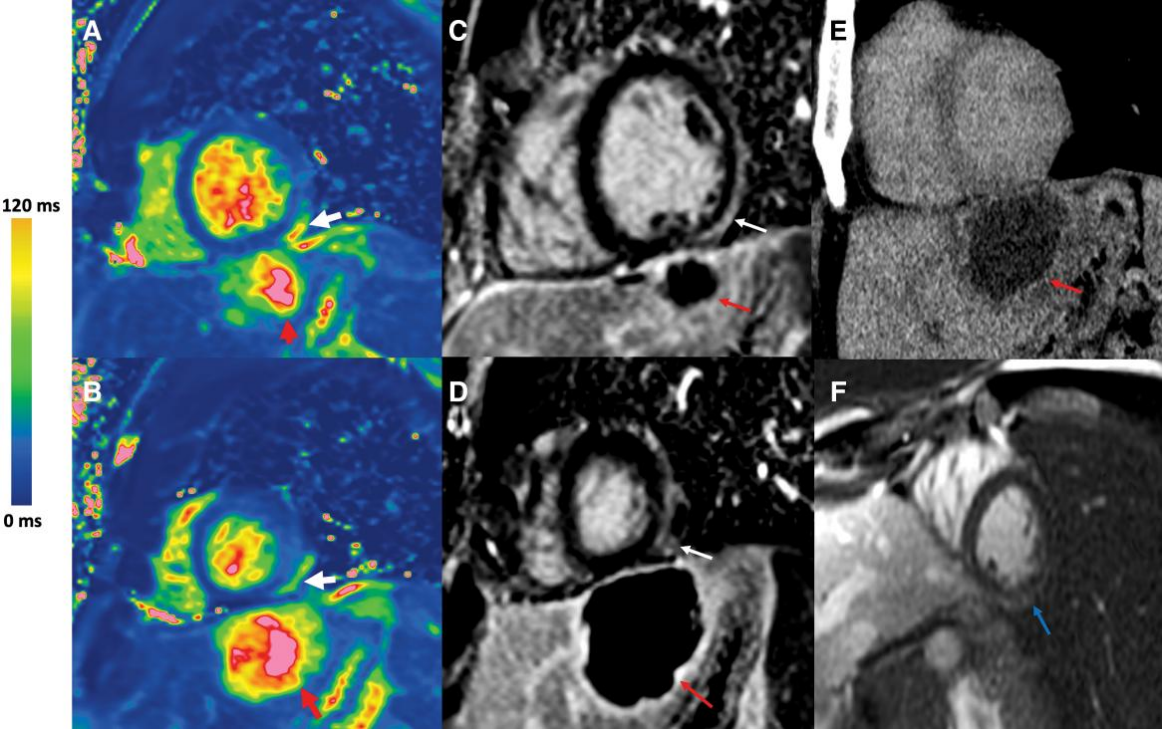
Pyogenic liver abscess with myocarditis. (*A* and *B*) T2 mapping sequence (CMR). Presence of a T2 elevation in the subepicardial region of the inferolateral wall (white arrow). Visualization of the hepatic abscess (red arrow). (*C* and *D*) LGE sequence (CMR). Presence of subepicardial enhancement in the inferolateral region (white arrow). Visualization of the hepatic abscess (red arrow). (*E*) CT scan showing hepatic abscess (red arrow). (*F*) Six-month CMR showing persistent LGE (blue arrow).

A 31-year-old male patient with no medical history presented with abdominal and chest pain with fever and significantly elevated troponin (610 ng/L, with a normal range being <20). He was hospitalized in the cardiac intensive care unit. The electrocardiogram was in sinus rhythm, with narrow QRS complexes, and no pronounced repolarization abnormality. The echocardiography did not reveal any abnormalities. The patient was referred for a CMR. T2 mapping sequence and LGE sequence are respectively shown in *Figure 1A* and *B* and *Figure 1C* and *D*. Diagnosis of myocarditis was made on myocardial T2 increase (measured at 65 ms, with normal values below 55 ms) and presence of LGE in the subepicardial region of the inferolateral wall (white arrows). The cardiac myocarditis involvement stemmed from a pyogenic left liver abscess located near the heart. This suspicion arose from the initial visualization on the CMR (red arrows), and it was subsequently confirmed through computed tomography (CT) (*Figure 1E*). After antibiotic treatment (amoxicillin/clavulanate 2 g three times a day for 6 weeks) and abscess drainage, the evolution was favourable. The identified pathogen was a *Fusobacterium*. Despite oral and digestive explorations including colonoscopy and endoscopy, the portal of entry was not found. The CMR at 6 months showed complete abscess regression, as well as the normalization of T2 with a persistent LGE scar (blue arrow) (*Figure 1F*).

### Patient 2

**Figure 2:**
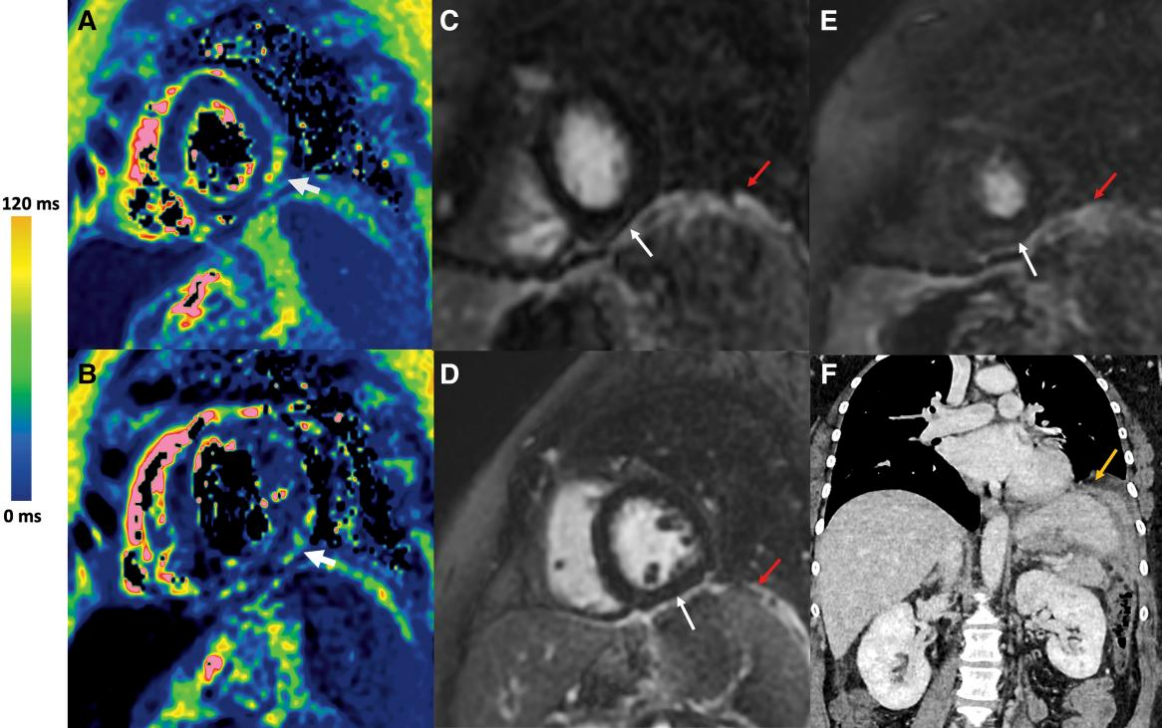
Myocarditis with peritoneal lymphoma carcinosis. (*A* and *B*) T2 mapping sequence (CMR). Presence of a T2 elevation in the subepicardial region of the inferolateral wall (white arrow). (*C*–*E*) LGE sequence (CMR). Presence of a subepicardial enhancement in the inferolateral region (white arrow). Visualization of a hyperintense thickening due to peritoneal lymphoma carcinomatosis (red arrow). (*F*) CT scan showing peritoneal lymphoma carcinomatosis (orange arrow).

A 54-year-old female patient had been under treatment for adult T-cell leukaemia/lymphoma since December 2021, having undergone six rounds of chemotherapy with CHOEP (cyclophosphamide, doxorubicin, etoposide, vincristine, and prednisone) and two rounds of chemotherapy with high-dose methotrexate until June 2022. Unfortunately, the patient showed tumoural progression with the development of peritoneal carcinomatosis. In mid-June, she presented with chest pain with significant troponin elevation (550 ng/L, with a normal range being <20). The electrocardiogram displayed the presence of negative T-waves in the inferior leads. The echocardiography did not reveal any specific anomalies. She underwent CMR in our institution: T2 mapping and LGE sequences are respectively shown in *Figure 2A* and *B* and *Figure 2C–E*. Diagnosis of myocarditis was based on T2 myocardial increase (measured at 62 ms, with normal values below 55 ms) and presence of discrete linear LGE in the subepicardial region of the inferolateral wall (white arrows). The loco-regional peri-splenic, peritoneal, and diaphragmatic hyperintense thickening processes due to lymphoma in contact of the heart were easily visualized on LGE sequence (red arrows). The diaphragmatic extension of peritoneal lymphoma carcinomatosis was then confirmed on thoraco-abdominal CT (orange arrow in *Figure 2F*). Another differential diagnosis to consider would have been cardiotoxicity, but this is not a typical effect of the medications used in this case. Given the substantial deterioration of her general condition, the lack of curative therapeutic options, and the poor prognosis of the disease, a decision was made to initiate palliative care.

### Patient 3

**Figure 3:**
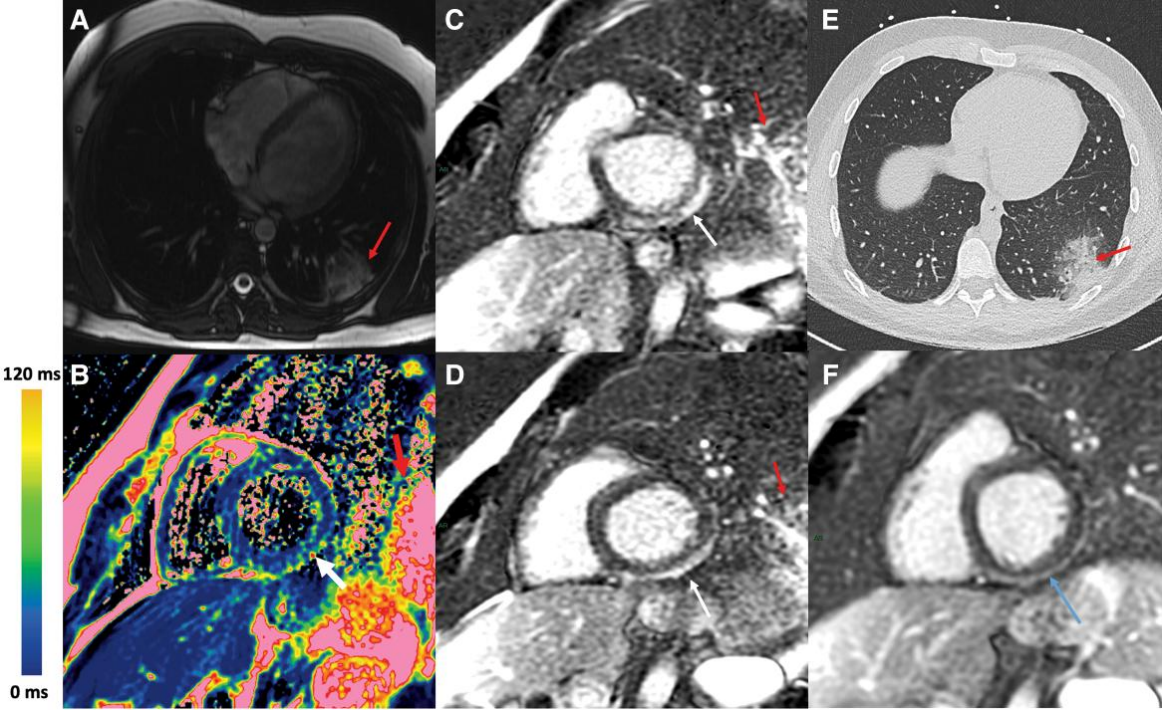
Myocarditis with pneumonia. (*A*) Trans-axial Fiesta localizer sequence (CMR). Presence of a left lower lobe consolidation (red arrow). (*B*) T2 mapping sequence (CMR). Presence of a T2 elevation in the subepicardial region of the inferolateral wall (white arrow). (*C* and *D*) LGE sequence. Presence of a subepicardial enhancement in the inferolateral region (white arrow). The left lower lobe consolidation can also be seen (red arrow). (*E*) CT scan showing left lower lobe consolidation. (*F*) Six-month CMR showing persistent LGE.

A 23-year-old male patient with no medical history experienced a 5-day history of influenza-like illness with cough and fever. Subsequently, the patient presented with retrosternal chest pain, which necessitated admission to the emergency department and subsequent transfer to the cardiac intensive care unit due to elevated troponin levels (6300 ng/L, with a normal range being <20). The electrocardiogram revealed millimetric ST-segment elevation in the inferior leads without reciprocal depression. The echocardiography detected moderate systolic dysfunction at 45% left ventricular ejection fraction, with more pronounced hypokinesis in the inferior wall. He underwent CMR in our institution. Trans-axial Fiesta localizer, T2 mapping, and LGE sequences are respectively shown in *Figure 3A* and *B* and *Figure 3C* and *D*. Diagnosis of myocarditis was made on T2 increase (measured at 64 ms, with normal values below 55 ms) and presence of LGE in the subepicardial region of the inferolateral wall (white arrow). The presence of acute pneumonia was detected using the trans-axial localizer, T2 mapping, and LGE sequences (red arrows). Cardiac CT did not reveal any coronary lesions and confirmed the pneumonia (*Figure 2E*) that supported the diagnosis of an infectious myocarditis associated with the pulmonary infection. Given the patient’s symptom history, the pneumonia appears to have preceded the myocarditis. No bacteria were identified, and the search for COVID-19 was negative. After antibiotic treatment (amoxicillin 1 g three times a day for 5 days) the evolution was favourable, and the patient was discharged after 5 days of hospitalization. The CMR at 6 months showed the persistence of LGE scar (blue arrow) without an elevation in T2 (*Figure 3F*).

## Discussion

Despite the advances in medical imaging and diagnostic techniques, the diagnosis and management of myocarditis remain challenging due to its variable aetiology and presentations. Guidelines recommend a multiparametric approach to diagnose myocarditis, combining clinical features, biomarkers, and imaging studies.^3,4,7^ Our case series supports this comprehensive approach, but more importantly, it highlights the role of CMR in diagnosing extra-cardiac aetiologies of myocarditis.

While myocarditis is commonly known for its systemic aetiologies, the concept of extra-cardiac causes remains underrepresented in the literature. The European Society of Cardiology guidelines recognize the importance of identifying the aetiology of myocarditis for optimal patient management, highlighting that the specific cause can significantly influence the treatment approach and prognosis.^7^ However, guidelines do not specifically address the role of CMR in diagnosing loco-regional aetiologies, an area that our case series sheds light upon.

The potential of CMR to detect loco-regional causes, as demonstrated in these cases, provides a new perspective on the diagnostic approach to myocarditis, even though such causes are rare. Beyond its role in visualizing myocardial inflammation, CMR’s broad field of view can capture nearby anatomical structures, providing crucial diagnostic clues that may be missed.^8^

Considering these findings, we propose that future guidelines could further emphasize the utility of CMR in exploring extra-cardiac causes. This could lead to more accurate diagnoses, appropriate treatments, and potentially improved outcomes for this subset of myocarditis patients.

## Conclusion

Our cases underline the necessity for a comprehensive, multidisciplinary approach to myocarditis diagnosis, encompassing the possibility of extra-cardiac aetiologies. We urge clinicians to stay vigilant for potential loco-regional causes, even when the clinical presentation primarily indicates myocardial involvement, and we call for further research in this underexplored area.

## Data Availability

The data underlying this article were accessed from Hôpital Européen Georges Pompidou, Assistance-Publique Hôpitaux de Paris (https://hopital-georgespompidou.aphp.fr). The derived data generated in this research will be shared on reasonable request to the corresponding author.

## Lead author biography

Hichem Sakhi is a cardiologist who has been certified since 2022. He completed a fellowship in cardiovascular imaging at the European Hospital Georges Pompidou in Paris, France. He has a particular interest in multimodal cardiac imaging.

**Consent:** All patients involved in the cases presented in this study provided informed consent for their medical details to be reported in an anonymized manner. They were informed about the purpose and their rights, including the right to withdraw consent at any time. After receiving this information, all patients voluntarily agreed to participate in the study and allowed their medical data to be used for research and publication purposes. Our article has been prepared in full compliance with the ethical guidelines established by COPE for patient consent statements.

**Funding:** None.
